# Impact of Physical Activity Interventions on High-Risk Pregnancies: A Systematic Review and Meta-Analysis

**DOI:** 10.3390/jpm14010014

**Published:** 2023-12-21

**Authors:** Cristina Silva-Jose, Michelle F. Mottola, Montse Palacio, Miguel Sánchez-Polán, Dingfeng Zhang, Ignacio Refoyo, Rubén Barakat

**Affiliations:** 1 AFIPE Research Group, Faculty of Physical Activity and Sport Sciences-INEF, Universidad Politécnica de Madrid, 28040 Madrid, Spain; cristina.silva.jose@upm.es (C.S.-J.); miguelsanpol@gmail.com (M.S.-P.); zhangdingfeng123@gmail.com (D.Z.); 2 R. Samuel McLaughlin Foundation-Exercise and Pregnancy Lab, School of Kinesiology, Faculty of Health Sciences, Department of Anatomy & Cell Biology, Schulich School of Medicine & Dentistry, Children’s Health Research Institute, The University of Western Ontario London, London, ON N6A 3K7, Canada; mmottola@uwo.ca; 3 Department of Maternofetal Medicine, Hospital Clínic (BCNatal-Fetal Medicine Research Center), Universitat de Barcelona, Fundació de Recerca Clínic Barcelona-IDIBAPS, 08036 Barcelona, Spain; mpalacio@clinic.cat; 4 Sports Department, Faculty of Physical Activity and Sport Sciences-INEF, Universidad Politécnica de Madrid, 28040 Madrid, Spain; ignacio.refoyo@upm.es

**Keywords:** pregnancy, restriction, bed rest, physical activity, outcomes

## Abstract

Pregnant women with absolute contraindications may be advised against physical activity throughout pregnancy. In this context, bed rest elevates the short-term risk of neonatal complications, thereby exacerbating negative long-term effects on childhood development. The aim of the current study was to investigate the impact of various physical activity interventions during bed rest or activity restriction in pregnancy on factors such as birth weight, preterm birth, maternal hypertension, gestational age at delivery, and the incidence of cesarean sections. Following the Preferred Reporting Items for Systematic Reviews and Meta-Analyses (PRISMA) guidelines, a systematic review was designed. The protocol was registered in the International Prospective Registry of Systematic Reviews (PROSPERO) (CRD42022370875). Nine studies, with a total sample of 3173 women, from six countries on four continents were included. There were significant differences in the relationship between bed rest status and birth weight (Z = 2.64; *p* = 0.008) (MD = 142.57, 95% CI = 36.56, 248.58, I2 = 0%, P_heterogeneity_ = 0.45) favourable to active groups. No significant differences were found in other analyzed outcomes. Pregnant women who experience this problem must maintain a minimum of daily activity to alleviate these physiological complications and the medical field must understand the consequences of physical inactivity during pregnancy.

## 1. Introduction

International guidelines recommend that healthy pregnant women should engage in a minimum of 150 min of moderate physical activity per week to maximize the recognized benefits of exercise during pregnancy, as supported by extensive scientific evidence [1]. However, it is important to note that women with absolute contraindications may be advised against physical activity throughout their entire pregnancy. In such cases, obstetric care providers may prescribe bed rest or restricted activity, either in a hospital or home setting, so as not to jeopardize the well-being of both the mother and the baby [2]. Although more recent guidelines would suggest that individuals with absolute contraindications may continue their usual daily activities but should not participate in more strenuous activities [3]. Many health care providers are not aware of the benefits of maintaining some type of physical activity, even with a high-risk pregnancy, and continue to prescribe bed rest or activity restriction [4].

Prescribing bed rest has historical roots tracing back to the ancient Greek physician Hippocrates [5]. Unfortunately, like many other medical practices, bed rest has become deeply ingrained in society. Consequently, activity restriction has been commonly prescribed by 90–95% of maternal healthcare professionals for both preventive and therapeutic purposes [6] for high-risk pregnancy complications (risk of miscarriage, preterm labor, preterm premature rupture of membranes, placenta previa, hypertension, or fetal growth restriction) even though it will only benefit approximately 20% of pregnant individuals [7,8,9,10,11,12]. Indeed, contrary to most medical prescriptions, compliance of such an indication is very high [13]. Unfortunately, prescription of activity restriction is often derived in its extreme form, which is complete bedrest.

This medical prescription has been strongly questioned from the year 2000 onwards. According to some authors, the practice of prescribing bed rest for pregnancies complicated by issues like preterm labor, intrauterine growth restriction, or hypertensive disorders does not seem to offer substantial benefits in terms of improving maternal and fetal outcomes [6,14]. Moreover, a significant drawback of bed rest lies in its restriction of various stimuli, including kinesthetic, tactile, auditory, visual, environmental, intellectual, emotional, and social interactions, which can have adverse consequences [15]. Therefore, complete bed rest has been linked to a range of unfavorable physiological and behavioral impacts on both maternal and fetal well-being, making it a notably distressing experience for women [16,17,18]. Consequently, the pursuit of enhancing maternal and fetal health outcomes in high-risk pregnancies characterized by conditions such as preterm labor, intrauterine growth restriction, or hypertensive disorders through the implementation of prescribing bed rest does not appear to yield favorable results [6].

During bed rest, a wide array of physiological disruptions affects the cardiovascular, respiratory, hematological, musculoskeletal, metabolic, immunological, thermoregulatory, and neuroendocrine systems [19,20,21]. Additionally, bed rest can lead to significant psychological and behavioral changes—including alterations in mood, cognition, and psychosocial well-being—often accompanied by heightened anxiety and mood disturbances. These multifaceted effects are significant side effects associated with the practice [22,23,24,25].

When considering the pregnant population, the imposition of activity restriction through bed rest during pregnancy can give rise to a range of adverse effects [26], including but not limited to:

Loss of muscle mass

Decreased lung volume

Nasal congestion

Constipation

Elevated risk of thromboembolism

Increased susceptibility to infections

Insulin resistance

Muscle discomfort and pains

Dizziness

Insomnia

Fatigue

Heightened bone resorption

Shortness of breath

Boredom

Difficulty concentrating

Increased family stress

Depression

In this context, bed rest elevates the short-term risk of neonatal complications, thereby exacerbating negative long-term effects on childhood development [6,12].

As a population stratum characterized by inherent risk factors, limited research has been conducted on physical activity in this population, largely due to the apparent nature of associated complications [27]. Nevertheless, in cases involving complications, the implementation of physical activity programs becomes challenging, especially when there is limited scientific literature available. Previous scientific evidence has demonstrated that physical activity can significantly mitigate the risk of thromboembolism, muscle strength decline, muscle atrophy, weight loss, dizziness, indigestion, bone loss, and heightened demineralization among high-risk pregnant women subjected to bed rest [22,24,25,28,29].

The intricate nature of the pregnancy process, especially in cases involving inherent risks, has led to a paucity of research dedicated to interventions aimed at mitigating the adverse effects of bed rest. Introducing an intervention that incorporates light physical activity may serve as a proactive approach to counteract the disruptions caused by bed rest during pregnancy. Nevertheless, there exists a significant knowledge gap on this topic within the realm of scientific literature. There is an urgent need to investigate the potential impact of alternative therapies, such as light physical activity, as a means to potentially alleviate the notable complications associated with bed rest during pregnancy [30].

The aim of the current study was to investigate the impact of various physical activity interventions during bed rest or activity restriction in high-risk pregnancy on factors such as birth weight, preterm birth, maternal hypertension, gestational age at delivery, and the incidence of cesarean sections.

## 2. Materials and Methods

Following the Preferred Reporting Items for Systematic Reviews and Meta-Analyses (PRISMA) guidelines, a systematic review was designed. The protocol was registered in the International Prospective Registry of Systematic Reviews (PROSPERO) (CRD42022370875).

### 2.1. Eligibility Criteria

The PICOS (population, intervention, comparison, outcome, and study design) strategy was used to lead this review with meta-analysis [31].

#### 2.1.1. Population

The population of interest were high-risk pregnant women older than 18 years of age regardless of their gestational age at the time of study admission with some type of physical activity restriction.

#### 2.1.2. Intervention

Interventions were based on prescribing some type of physical activity or following a normal lifestyle in those diagnosed with a high-risk pregnancy. 

#### 2.1.3. Comparison

The comparison included any form of hospital rest or physical activity restriction to improve some maternal-fetal health parameter compared to daily activity. Co-interventions are also recorded: bed rest combined with other interventions (e.g., pharmacological intervention).

#### 2.1.4. Variable

The primary study variables were birth weight and preterm birth. The studies had to contain at least the primary study variable registered for analysis, or, failing that, registered as a potentially relevant secondary variable for analysis. The secondary variables were maternal hypertension, gestational age at delivery, and C-sections.

#### 2.1.5. Study Design

We selected studies pertaining to interventions, including randomized clinical trials, quasi-experimental clinical trials, and feasibility randomized clinical trials. However, observational studies, various types of reviews (systematic, narrative, or systematic reviews with meta-analysis), and qualitative research were excluded from our analysis.

### 2.2. Data Sources

The following databases were exhaustively reviewed: Web of Science, Scopus, Sport Discus, Academic Search Premier, MEDLINE, ERIC, OpenDissertations, Clinicaltrial.gov, and Cochrane Database of Systematic Reviews through the portal of the Universidad Politécnica de Madrid. 

Between October 2022 and November 2022, the search was performed. To guarantee equality, the same article selection criteria was used for all the databases, controlling vocabulary and syntax selection. Articles written between 1975 and 2022 written in Spanish and English were considered. The bibliographic references of selected studies were reviewed to identify other potentially selectable studies that could be discarded in the first search.

### 2.3. Selection and Data Extraction

To identify potential papers in accordance with our inclusion criteria, two investigators independently (CS and MS) reviewed the titles and abstracts retrieved through electronic searches. Once abstracts passed the initial screening, we conducted full-text searches. For those results selected for data extraction, full texts were independently examined. To ensure no critical information was overlooked, pertinent data were also gathered from all registries. Both researchers reached a consensus to determine the inclusion of studies in cases where one of them had initially suggested exclusion. In instances of complete disagreement, a third researcher (DZ) provided their criteria for study inclusion. One individual (DZ) collected the information required to populate the tables, which were subsequently independently reviewed by a subject matter expert to assist in further analysis (CS). Except for papers presenting data in graphical form, straightforward methods were employed to extract data from tables or the text. This approach ensures the reliability and authenticity of the data.

The extracted information encompassed study characteristics (including author’s name, country, and publication year), article type (randomized clinical trial), sample size, group differences, and intervention/exposure (exercise recommendation and/or measurement) as well as details regarding frequency, intensity, time, and type of exercise; supervision of the intervention; duration; and adherence to the intervention. Additionally, we captured information on primary and secondary variable(s) analyzed and any co-interventions if present (Table 1).

### 2.4. Evidence Quality Assessment

The Grading of Recommendations Assessment, Development, and Evaluation (GRADE) methodology was used to rate the quality of the evidence for each research design and primary outcome. This framework offered a systematic and standardized method to evaluate the evidence across various research [32]. This meta-analysis included 6 highly rated studies.

### 2.5. Risk of Bias Assessment

The assessment of bias risk was conducted in accordance with the *Cochrane Handbook* Potential biases were scrutinized in each study, encompassing attrition bias (related to incomplete follow-up and high loss during follow-up), selection bias (associated with inadequate randomization procedures in RCTs/interventions), performance bias (pertaining to intervention compliance in RCTs/interventions), detection bias (relating to faulty outcome measurement), and reporting bias (involving selective or incomplete reporting of results) [33].

### 2.6. Publication Bias Assessment

In order to assess potential publication bias in each developed meta-analysis, the Egger regression test was employed due to its enhanced sensitivity in detecting publication bias under conditions of weak or moderate heterogeneity. Typically, this test yields a metric indicating significant publication bias when *p* < 0.1 [34].

### 2.7. Statistical Analysis

Statistical analysis was performed using RevMan V.5 software. The overall confidence interval (CI) for the continuous outcome, birth weight, as extracted from medical records in the considered articles, was calculated utilizing the mean difference (MD) [35]. In continuous analyses, a weighted approach that factored in the sample size or the number of events provided by each study was employed to determine the adjusted average. This weighting method allowed for a more precise representation of the entire dataset by accounting for the varying contributions of information from each study.

The cumulative odds ratio (OR) was computed using a random effects model [34]. For all continuous outcomes derived from the medical records of the analyzed articles, MD was utilized to calculate the overall confidence interval (CI) as well [35].

To assess the extent of variability in the outcomes, we calculated the I2 statistic. This metric offers insights into the proportion of variation in the observed intervention effect among studies that can be attributed to heterogeneity rather than random chance. The interpretation of the I2 statistic followed established thresholds: 25% for low heterogeneity, 50% for moderate heterogeneity, and >75% for high heterogeneity [36].

**Table 1:** Characteristics of selected studies.

Refs.	Author	Year	Country	Type	N	NA	BR	Normal Activity	Bed Rest	Principal Outcomes	Secondary Outcomes	Co-Intervention
[37]	Bigelow et al.	2015	USA	RCT	36	18	18	No limitation on their activity and recommendation of walking for a minimum of 20 min at least three times a day and they had permission for doing all activity as desired.	Spend most of their day in their hospital bed, usually in a reclined or sleeping position.	Amniotic fluid volume changes and latency to delivery after premature rupture of membranes.	Maternal outcomes and neonatal outcomes.	NO
[38]	Brun et al.	2011	Canada	Feasibility RCT	11	6	5	Muscle-conditioning exercises were performed using the resistance tool with enough resistance for the subject to perform 2 sets of 15 repetitions in a side-lying position or in a 45-degree recumbent recline. Total intervention time was 30 min.	The women in the control group listened to the same music as those in the exercise group while in either a side-lying position or a 45-degree recumbent recline. Total time for the complete bed rest and music intervention was 30 min.	HR, BP, and uterine contraction.	Birth weight.	NO
[39]	Crowther et al.	1992	Zimbabwe	RCT	218	108	110	Continue normal activities at home and no restrictions were advised.	Rest in bed as much as possible, although voluntary ambulation around the ward was allowed.	Proteinuria.	Maternal outcomes and neonatal outcomes.	NO
[40]	Elliot et al.	2005	USA	RCT	73	36	37	Continue normal activities, including home and work responsibilities.	Bathroom and showering privileges.	Gestational age Preterm Labor Management.	Preterm birth rate, low and very low birthweight, (LBW, VLBW), and neonatal intensive care unit (NICU) days.	Magnesium sulfate (MgSO_4_)
[41]	Hobel et al.	1994	USA	RCT	2654	1774	880	Traditional social work counseling and stress reduction and relaxation techniques.	The bed rest program consisted of advising women to rest three times a day for an hour and to keep a log of the rest periods and any contractions felt by self-palpation during the rest periods.	Preterm delivery rate.	Maternal outcomes.	Oral progestin (Provera)
[42]	Leung et al.	1998	Hong Kong	RCT	88	44	44	Continue normal activities.	Rest in bed as much as possible.	Hypertension and proteinuria.	Maternal and neonatal outcomes.	NO
[43]	Martins et al.	2019	Portugal	RCT	32	18	14	Walks to the ward canteen and had full bathroom privileges.	Complete bed rest were kept in antepartum confinement to bed and restricted to bedpan use.	Latency time and chorioamnionitis incidence.	Indication for delivery, mode of delivery, thromboembolic events, placental abruption, cord prolapse and fetal demise, and neonatal outcomes.	NO
[44]	Mathews et al.	1977	United Kingdom	RCT	135	64	71	Continue normal activities including housework and shopping.	Bed rest in whatever position was most comfortable for them for most of the time but allowed up to meals and toilet.	Renal function and feto-placental wellbeing, and	Maternal and neonatal outcomes	Phenobarbitone (15 mg)
[45]	Mathews et al.	1982	United Kingdom	RCT	40	20	20	Moving freely about the hospital ward.	Complete bed rest.	fetoplacental well-being and fetal outcome.	Serum human placental lactogen (ihPIz), oestriol concentrations, maternal and neonatal outcomes.	NO

Refs.: references. Author: last name. Year: year of publication. Country: country in which the study was been done. Type: type of study; N: total number of women included in the analysis. NA: number of women doing normal activity included in the analysis. BR: number of women in bed rest included in the analysis. RCT: randomized clinical trial.

## 3. Results

### 3.1. Study Selection

**Figure 1:**
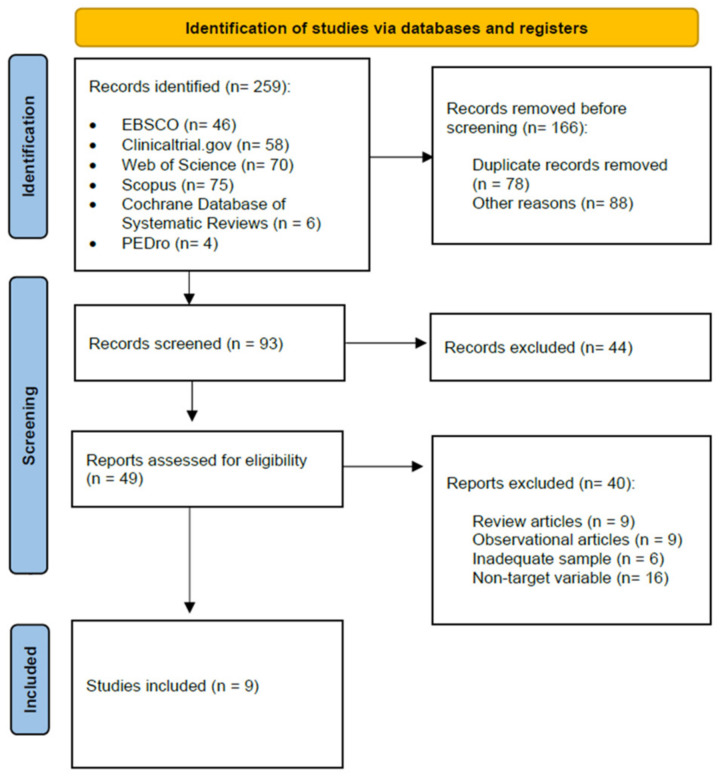
Flow chart of the review process.

Figure 1 displays the PRISMA diagram, which provides an overview of the search results along with explanations for exclusions. 

### 3.2. Quality of Evidence

**Figure 2:**
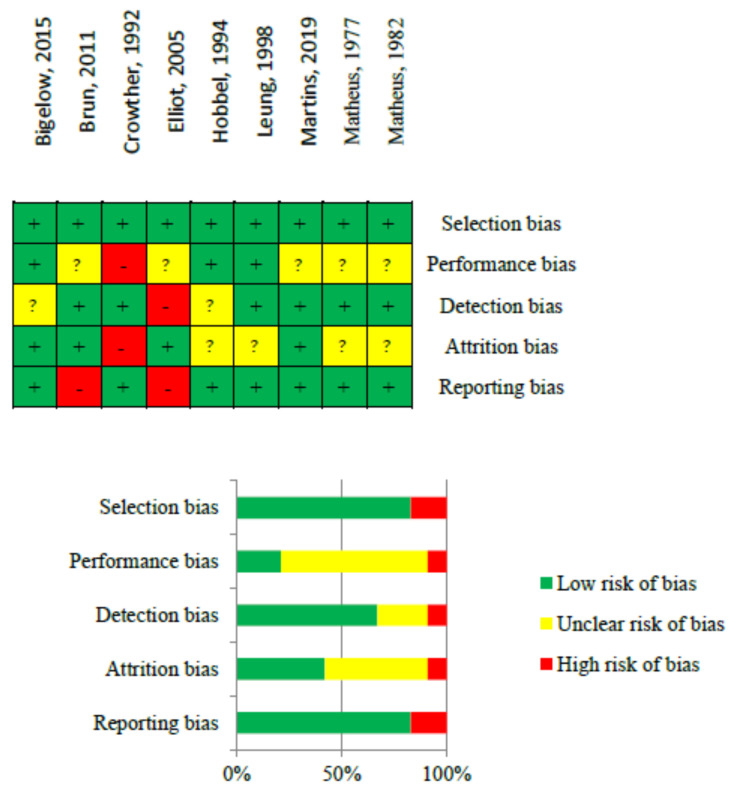
Risk of bias of selected studies [37,38,39,40,41,42,43,44,45].

The overall quality of evidence across studies ranged from poor to excellent. Despite the identification of bias risks, it was determined not to exclude any papers from the analyses (Figure 2). Reviewing sources of bias, most of the studies presented a low risk of bias on selection, detection, and reporting bias. Some of the studies presented an unclear performance risk of bias due to the blinding of participants in this type of intervention being complicated.

### 3.3. Study Characteristics

In total, we analyzed nine studies that met the inclusion criteria, encompassing a collective sample size of 3173 women from six countries across four continents, as summarized in Table 1. Among these nine studies, four were conducted in North America, specifically in the United States [37,40,41] and Canada [38]. The five remaining studies were distributed globally, with two conducted in the United Kingdom [44,45] and one each in Portugal [43], Zimbabwe [39], and Hong Kong [42].

Among the selected studies, eight were randomized clinical trials, while one was a feasibility randomized clinical trial. Within the articles identified, three also incorporated pharmacological co-interventions [40,41,44].

Due to the unique nature of this research, two distinct types of studies have been identified. Firstly, there are eight articles that, in the context of bed rest or activity restriction, permit women to continue their daily routines [37,39,40,41,42,43,44,45], with one of them specifically recommending a 20 min walk three times a day [37]. On the other hand, one article was identified that prescribed a form of exercise to women undergoing bed rest in comparison to a control group. The prescribed exercises involved strength training, specifically 2 sets of 15 repetitions of muscle-conditioning exercises using elastic bands, for a duration of 30 min [38]. Additional details are presented in Table 1.

The primary study variables focused on physiological elements associated with high-risk pregnancies. In contrast, the secondary variables encompassed maternal outcomes—including demographic characteristics (such as age, parity, race, marital status, education, gravidity, maternal height and weight, hypertension, use of antihypertensive therapy, or other high-risk factors)—as well as prenatal outcomes (gestational age at delivery, mode of delivery, induction of labor, abruption, proteinuria, severe hypertension, endometritis, chorioamnionitis, and thromboembolic events).

Additionally, neonatal outcomes were examined, including birth weight; low birth weight; small-for-gestational-age status; preterm birth; APGAR scores at 1 and 5 min; neonatal intensive care unit admission; length of postnatal hospital stay; neonatal sepsis; and neonatal pulmonary, cardiac, and renal outcomes. Stillbirth or neonatal death and other related morbidities were also considered.

The analysis of primary and secondary outcomes is presented in the following paragraphs.

### 3.4. Activity vs. Physical Activity Restriction during Pregnancy on Birth Weight

**Figure 3:**
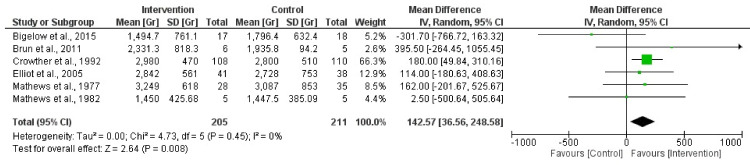
Effect of activity in high-risk pregnancy compared to activity restriction on birth weight [37,38,39,40,44,45].

A total of six randomized clinical trial studies were incorporated in this analysis [38,39,40,41,44,45]. The results revealed statistical significance in the relationship between the bed rest condition and birth weight (Z = 2.64; *p* = 0.008) (MD = 142.57, 95% CI = 36.56, 248.58, I2 = 0%, P_heterogeneity_ = 0.45). Figure 3 depicts the forest plot corresponding to the conducted meta-analysis. Quantification evaluation of the risk of publication bias test in the analyzed articles showed that there was no potential publication bias (*p* = 0.95) in this analysis.

### 3.5. Activity vs. Physical Activity Restriction during Pregnancy on Preterm Birth

**Figure 4:**
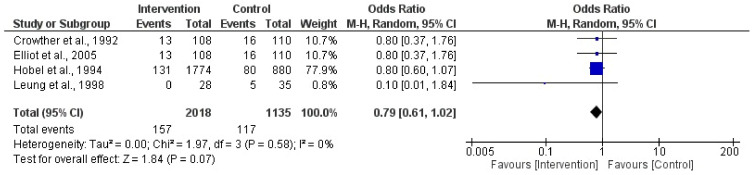
Effect of activity in a high-risk pregnancy compared to activity restriction on preterm birth [39,40,41,42].

In this analysis, a total of four randomized clinical trial studies were incorporated [40,41,42,43]. No significant relationship was found (Z = 1.84; *p* = 0.07). The results showed that there are no associations between bed rest or physical activity restriction and preterm birth (OR = 0.79, 95% CI = 0.61, 1.02, I2 = 0%, P_heterogeneity_ = 0.58). The quantitative assessment of publication bias risk in the analyzed articles indicated the absence of potential publication bias (*p* = 0.82) in this analysis. Figure 4 illustrates the forest plot corresponding to the conducted meta-analysis.

### 3.6. Activity vs. Physical Activity Restriction during Pregnancy on Hypertension

**Figure 5:**
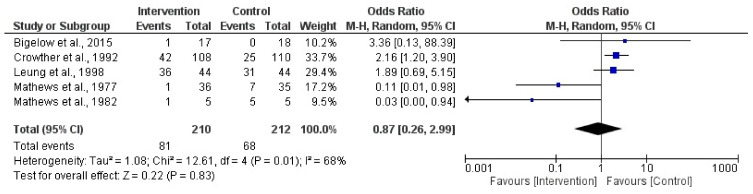
Effect of activity in a high-risk pregnancy compared to activity restriction on maternal hypertension [37,39,42,44,45].

In this analysis, a total of 5 randomized clinical trial studies were incorporated [38,40,43,44,45]. The results showed no association between bed rest or physical activity restriction and maternal hypertension (Z = 0.22; *p* = 0.83) (OR = 0.87, 95% CI = 0.26, 2.99, I2 = 68%, P_heterogeneity_ = 0.01) Figure 5 depicts the forest plot corresponding to the conducted meta-analysis. Quantification evaluation of the risk of publication bias test in the analyzed articles showed that there was potential publication bias (*p* = 0.04) in this analysis.

### 3.7. Activity vs. Physical Activity Restriction during Pregnancy on Gestational Age at Delivery

**Figure 6:**
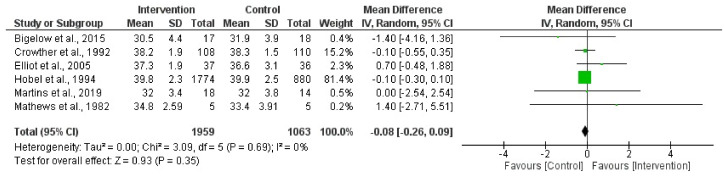
Effect of activity in a high-risk pregnancy compared to activity restriction on gestational age at delivery [37,39,40,41,44,45].

In this quantitative analysis, a total of six randomized clinical trial studies were incorporated [38,40,41,42,43,45]. The results report no association between bed rest or physical activity restriction and gestational age at delivery (Z = 0.93; *p* = 0.35) (MD = −0.08, 95% CI = −0.26, 0.09, I2 = 0%, P_heterogeneity_ = 0.69). Figure 6 depicts the forest plot corresponding to the conducted meta-analysis. Quantification evaluation of the risk of publication bias test in the analyzed articles showed that there was no potential publication bias (*p* = 0.36) in this analysis.

### 3.8. Activity vs. Physical Activity Restriction during Pregnancy on C-Section

**Figure 7:**
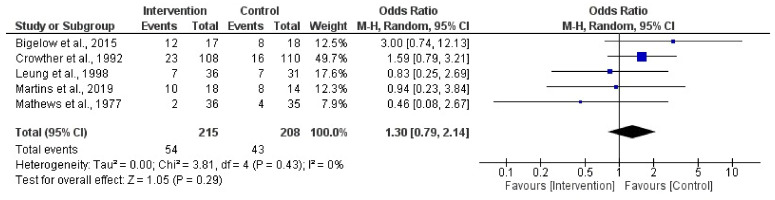
Effect of activity in a high-risk pregnancy compared to activity restriction on C-section [37,39,42,44,45].

In this analysis, a total of five randomized clinical trial studies were incorporated [38,40,43,44,45]. The results showed no association between bed rest or physical activity restriction and C-section (Z = 1.05; *p* = 0.29) (OR = 1.30, 95% CI = 0.79, 2.14, I2 = 0%, P_heterogeneity_ = 0.43). The quantitative assessment of publication bias risk in the analyzed articles indicated the absence of potential publication bias (*p* = 0.32) in this analysis. Figure 7 illustrates the forest plot corresponding to the conducted meta-analysis.

### 3.9. Activity vs. Physical Activity Restriction during Pregnancy on Other Perinatal Outcomes

Potentially analyzable outcomes were identified in the articles that met the criteria, such as maternal blood pressure, type of delivery, latency to delivery, NICU admission, and neonatal death. However, they could not be assessed due to the inconsistency and limited record of the variables.

## 4. Discussion

As far as we know, the present study is the first systematic review to examine the influence of a physical activity intervention on high-risk pregnancies, analyzing birth weight and other perinatal variables, based on nine studies (3173 pregnancies). This work provides a key contribution and starting focus for the performance of physical activity in complicated pregnancies instead of total restriction of physical activity or bed rest conditions. At this point, it was observed that maintaining physical activity increases the probability of more suitable birth weights and limited preterm birth in high-risk women. 

Our systematic review with meta-analysis examined the relationship between bed rest during pregnancy and birthweight and preterm birth, suggesting that lower birth weights were found in the those who limit their physical activity or stay with bed rest limitations. These results are consistent with previous studies that suggest that bed rest can decrease maternal weight gain and be associated with an increased risk of fetal growth restriction [45]. Additionally, gestational age at delivery is not improved with activity restriction [12].

There is little general evidence to support the policy of routine hospital admission for bed rest and even less about the possible beneficial effect on fetal growth of hospital bed rest for pregnant individuals [23]. Similarly, in other reviews, it has been suggested that a continuous restriction of activity does not improve gestational hypertension [12]. There appears to be no added benefit in restricting activity, as the Society of Obstetricians and Gynecologists of Canada does not recommend activity restriction for the treatment of non-severe preeclampsia [27].

Analysis of the secondary study variables (hypertension, gestational age at delivery and c-sections) showed that bed rest did not provide an added benefit in limiting these complications. This would suggest that maintaining regular daily physical activity during pregnancy does not pose any added risk compared to absolute rest. Consequently, expectant mothers need to maintain a healthy lifestyle throughout their pregnancy. However, it is important to consider the results of the reviewed studies in a comprehensive way to make individualized decisions for each specific case. In addition, more research is needed to understand the minimum and maximum influence of physical activity levels in unfavorable pregnancy conditions.

When physical exercise programs are examined in the scientific literature regarding those who must remain on bed rest while pregnant, results are outdated and scarce. For the current review, three studies were found with different objectives, methodologies, and interventions, although the central axis of these programs was strength exercises. Interventions are of short duration, and only acute short-term effects were examined. Like the study by Brun et al. [38] two other interventions discussed in the current review used strength exercises with different methodologies and different levels of activity within the interventions: 30 min of exercises with elastic bands for 4 days of hospitalization [6] and an average intervention of 6 sessions of 1 h of exercise, 3 days a week of resistance training in an aquatic environment [46]. However, studies that include physical exercise as an agent to mitigate the negative effects of bed rest—such as fatigue, weakness, joint pain, cardiovascular deterioration, and increased postpartum recovery [14]—are scarce, so there is an urgent need for research.

It is important to consider that the data analyzed by the current systematic review and meta-analyses have several limitations, such as heterogeneity, publication bias, or low quality of evidence. Due to the different methodologies used, as well as the disparity in the recorded variables, the conclusions about this situation during pregnancy are limited. 

Previous literature suggests that maintaining a normal physical activity routine or performing some physical exercise while on bed rest is safe and does not add risk to the particular medical condition. However, data are limited, and this should serve as a starting point and motivation for future research. On the other hand, the next level is that if bed rest is needed because of a particular severe condition (intravenous, continuous monitoring…), some activity could be performed while on bed rest, which is a field of research to be addressed in the future.

Similarly, the articles analyzed show a high difference in years of publication. Since 1978, there have been four systematic reviews that addressed the role of activity restriction in the prevention of maternal and neonatal complications, reaching conclusions similar to those of the current review [47,48,49], suggesting that bed rest would not help in obtaining better perinatal outcomes. For this reason, high-quality studies are needed to provide more robust clarity regarding the impact of exercise during pregnancy in different maternal–fetal parameters and the role of physical activity in mitigating side-effects of activity restriction.

Unfortunately, the small number of studies may limit important conclusions, as this is a complex area. For this reason, emphasis should be placed on the importance of exercise to reverse the physiological effects of inactivity. In conclusion, pregnant individuals who experience this complex problem must maintain a minimum of daily activity to mitigate these physiological effects and the medical field must understand the consequences of physical inactivity during pregnancy. For this reason, studies focused on the influence of sedentary behavior on maternal–fetal health parameters in high-risk pregnancies are necessary. In addition, the benefits of exercise on psychosocial health to improve physical conditioning postpartum and when to resume activity have yet to be studied.

## Data Availability

No new data have been reported.
